# Are Lessons Learned in Setting Cut Points for Detection of Anti-Drug Antibodies Also Useful in Serology Assays for Robust Detection of SARS-CoV-2 Reactive Antibodies?

**DOI:** 10.1208/s12248-020-00510-8

**Published:** 2020-10-06

**Authors:** Ronald R. Bowsher, Viswanath Devanarayan

**Affiliations:** 1 B2S Life Sciences llc, 97 East Monroe Street, Franklin, Indiana 46131 USA; 2 GlaxoSmithKline, 1250 South Collegeville Road, Collegeville, Pennsylvania 19426 USA; 3 University of Illinois at Chicago, 1200 W. Harrison Street, Chicago, Illinois 60607 USA

**Keywords:** COVID-19, cut point, diagnostics, immunogenicity, serology, statistics

The current COVID-19 global pandemic has generated widespread interest across clinical, research, academic, and governmental laboratories, as well as at Biopharma companies for the application of *in vitro* diagnostic assays to detect the presence of SARS-CoV-2 virus or to characterize the emergence of an adaptive immune response against this virus. Following declaration of the COVID-19 public health emergency by Alex Azar, Secretary of Health and Human Services, on Jan. 31, 2020, the FDA issued an “immediate-in-effect” guidance on 29 February to make *in vitro* diagnostic tests available by Emergency Use Authorization (EUA) to address the urgent need for IVD tests to support diagnosis and treatment of COVID-19 infections (1). Subsequently in late April, the FDA released the umbrella EUA guidance to offer an additional route for expediting approval and market availability of serological tests for COVID-19 (2).

Unlike molecular diagnostic and viral antigen tests that detect an active viral infection, serological assays detect serum antibodies to SARS-CoV-2 viral antigens in individuals who have exhibited an adaptive immune response as part of either an active or prior infection (3). As such, serology tests offer the potential to verify that individuals, who had a prior SARS-CoV-2 infection with clinical symptoms or who have remained asymptomatic, developed a humoral antibody response. Despite their perceived value, commercial serology assays, point-of-care devices, and homebrew laboratory developed tests (LDTs) vary appreciably with respect to their design attributes and performance capabilities. Consequently, serological assays are known to demonstrate inconsistency in antibody detection due to differences in their clinical sensitivity and specificity (4). For example, recent evidence suggests that some assays may be prone to false-positive results due to the presence of serum antibodies against other coronaviruses that are also cross-reactive to structurally homologous epitopes present in the SARS-CoV-2 virus (5–7). While at present the overall value of serological testing remains unclear (8), this technology will undoubtedly find broad application in epidemiological surveillance studies, contact tracing, and in evaluating antigen-specific humoral immunity after active immunization (4,9,10).

Currently, EUA-approved serological tests include high complexity ELISA designs, moderate complexity instrumentation-based tests, and lateral flow point-of-care devices. Because the reliability of serological assays remains a topic of concern to both the scientific community and general public, the FDA issued an updated policy on May 4, 2020 that required antibody test manufacturers to submit an EUA request within 10 business days (1). Shortly thereafter on May 21, approximately 50 tests were removed from the EUA approved list either because of a failure to submit data on time or due to technical concerns (3). By mid-August, the number of serology assays removed from the EUA approved list increased to 97, while the number of approved commercial tests is currently 37 (11,12). Continued vigilance for evaluation of assays on the EUA-approved list should help ensure that the reliability and value of serological testing will increase over time.

During our development of a direct-binding immunoassay that employs SARS-CoV-2 trimer spike protein as the capture antigen (13), we noted a striking similarity between a serology assay that is used in a CLIA-certified lab for detection of SARS-CoV-2-reactive serum antibodies and those that are used widely in the Biopharma industry and CROs for detection of anti-drug antibodies (ADA) in support of immunogenicity assessments of biotherapeutic drugs. Accordingly, we prepared Table I to highlight the similarities and differences between these two different categories of antibody assays. Upon inspection, it is readily apparent that some practices differ between these two testing paradigms due to differences in their intended applications. Nonetheless, some analytic performance characteristics are common, including the requirement for data-driven determination of a screening cut point (SCP) to classify serum results as either being potentially positive or negative for the presence of reactive antibodies.

**Table I:** Comparison Of Analytical Approaches Used For Antibody Detection

Characteristic	Anti-drug antibody assays	*SARS-CoV-2 serology assays
Analytical objective	Detect emergence of a treatment-induced humoral AB response upon repeated administration of a biotherapeutic	Detect presence of reactive serum antibodies to SARS-CoV-2 viral antigens
Testing purpose	Support risk assessment to evaluate the immunogenic potential of a therapeutic in patients	To evaluate if a patient has developed an adaptive immune response to SARS-CoV-2 virus
Testing population	Defined by nonclinical or clinical study protocol	General public
Regulatory oversight	Oversight by FDA with lab work conducted under GCP/GLP bioanalysis as outlined in guidance documents with the most recent being published in Jan. 2019 (14)	The Centers for Medicare and Medicaid Services (CMS) regulates all clinical lab testing performed on humans in the USA through Clinical Lab Improvement Amendments (CLIA), part of the Public Health Service Act. CLIA covers about 260,000 labs. CLIA-certified Lab/CAP or other proficiency testing program
Where is testing performed?	Biopharma lab and CROs	CLIA-certified lab or locally using a point-of-care device
When is testing performed?	During nonclinical and clinical development. Also, post-approval	Whenever individuals seek diagnostic verification for the development of an adaptive immune response to the SARS-CoV-2 virus
Reported result	POS/NEG, titer (dilution only) Neutralizing YES/NO, cross-reactivity to endogenous and domain specificity	IVD specific, usually POS/NEG. Sometimes quantitative ng/mL
Assay design?	Most often bridging or antigen direct binding assay	Typically, a direct binding assay design involving antigen capture
Testing paradigm	Industry harmonized tiered testing with screening, confirmation, titer, and neutralizing antibody testing	Screening assay with IVD having an assigned protocol CP or LDT with internal lab established CP
Criteria for cut point assignment	FDA specifies that Screening assay CPs will have a 5% FPER to lessen risk of a false-negative outcome	Mostly an internal process in clinical diagnostic labs for LDT that is performed according to CLSI or other. IVD industry lacks a harmonized process for statistical CP assignment.
Assay run acceptance	Mostly standardized across labs. Criteria can vary lab-to-lab and governed by SOPs.	Governed by CLSI and IVD protocol
Sensitivity	Determined with POS. surrogate anti-therapeutic AB. 100 ng/mL is required threshold for detecting a clinically meaningful ADA level	Ability to detect the presence of reactive antibody in a sample that is truly positive (i.e., sample is from patient who is confirmed POS. for COVID-19 by molecular or antigen-based test). Includes 95% confidence interval
Specificity	Refers to the ability of an assay to exclusively detect the target analyte (i.e., antibody). Typically evaluated empirically by competitive binding experiments. May pertain to AB cross-reactivity to a structurally related homolog, endogenous entity or an unique structural domain in a therapeutic	Failure to detect the presence of reactive antibody in a sample that is truly negative (i.e., sample was collected from patient prior to COVID-19 pandemic). Includes 95% confidence interval
Positive predictive value (PPV)	Not applicable	Likelihood that a POS test result is truly positive for the presence of SARS-CoV-2 antibodies. Depends heavily on disease prevalence and assumed to be 5% (see Table II)
Negative predictive value (NPV)	Not applicable	Likelihood that a NEG test result is truly negative for the presence of SARS-CoV-2 antibodies. Depends heavily on disease prevalence and assumed to be 5% (see Table II)
Drug tolerance	Important to assess degree of interference from administered drug to lessen risk for reporting a false-negative result	Not applicable
Target interference	Important to assess for assays involving monoclonal antibodies due to false positive	Not applicable
Precision	Specified in FDA guidance. In most cases the intra- and inter-assay precision requires CV of ≤ 20% for replicate measurements	Governed by CLSI or specified in an IVD protocol

*www.fda.gov/medical-devices/emergency-situations-medical-devices/eua-authorized-serology-test-performance/

While publications and regulatory guidance documents for clinical *in vitro* IVDs and point-of-care (POC) devices recommend the establishment of a reliable detection cutoff for identifying antibody-positive samples (15–19), a consensus statistical-based approach for setting cut points appears to be lacking based on a perusal of published literature. Insight into setting of cut points for CLIA serological assays is complicated further by the proprietary nature of most commercial IVDs and POC devices in which kit documentation lacks detail about the approach that was used for setting the assay’s cut point. Rather, emphasis is usually placed on justifying a diagnostic cutoff based on the analysis of a receiver operating characteristic (ROC) curve of pilot studies with clinical samples. This cut point approach basically sets the threshold retrospectively based on observed results of a developed assay.

In contrast, much progress has been made over the past two decades within the Biopharma industry to establish a consensus data-driven approach to support immunogenicity testing for detection and characterization of anti-drug antibodies (14,20–23). This circumstance prompted us to question the potential value of applying lessons learned in setting cut points for anti-drug antibody assays to IVD and LDT methods. We believe that the application of a similar cut point strategy to the one that is used for detecting ADA could have value for aiding reliable detection of SARS-CoV-2 serum antibodies by CLIA diagnostic assays.

Today’s multi-tiered ADA testing approach includes standardized recommendations for statistical determination of cut points for screening (Tier 1), confirmation (Tier 2), titer assessment (Tier 3), and neutralizing antibody testing (Tier 4) (14,20–23). For development of a screening cut point during assay validation to identify ADA-positive samples for a clinical trial, the generalized approach involves testing of around 50 presumptive antibody-negative samples across 6 assay runs (3 assay runs × 2 analysts) to yield about 300 total observations which are analyzed statistically after removal of analytical and biological outliers to compute a cut point with a 5% false-positive error rate (FPER). To lessen the degree of analytical variability, the common approach is to use a negative control serum pool to normalize the assay signal responses. Application of the 5% FPER is recommended in immunogenicity guidance documents from the FDA and EMA to avoid failure to detect a low level of clinically meaningful antibody, an undesired false-negative outcome (type II error), due to potential consequences for patient safety or treatment efficacy (14,23).

The intended purpose for detection of SARS-CoV-2-reactive antibodies in CLIA assays differs from ADA testing. In this circumstance, a positive antibody result may be used to judge whether someone can interact freely in a high-risk environment, with senior citizens, with individuals who have compromised immunity or for making a decision about whether to travel and intermingle in crowded environments. Consequently, accurate detection of antibodies in serology assays places greater importance on limiting the FPER (i.e.*,* higher specificity), a type I error, relative to the false-negative error rate (FNER). This is because the consequence of incorrectly classifying individuals who are truly antibody negative as being antibody positive is more severe than incorrectly categorizing those that are truly antibody positive as being antibody negative. Similarly, the positive predictive value (PPV) is relatively more important than the negative predictive (NPV), as it is more critical to ensure that those who are identified analytically as being positive are truly positive.

From the illustrations in Table II, assuming 5% prevalence of SARS-CoV-2-reactive antibodies in the general population, and keeping the FNER at 5% (i.e.*,* 95% sensitivity), we discover that setting the cut point at a 5% FPER (i.e.*,* the threshold used routinely in an ADA screening assay (95% specificity)) will result in a PPV of only 50%. That is, only half of the individuals who are identified by testing as being positive are truly antibody positive! If, however, the FPER is reduced to 1%, the PPV increases to 83.33%. While this PPV is much better, it is still inadequate if a million people or more are tested in a population with 5% disease prevalence (i.e., 9500 individuals will be identified incorrectly as antibody positive which puts too many individuals at increased risk for misclassification). Further reduction of the FPER to 0.1% increases the PPV to 98.04%! Even further lowering of the FPER to only 0.01% results in 99.8% PPV (Table III), which translates to only 95 out of a population of 1 million as being incorrectly identified as antibody positive.

**Table II:** Impact Of Using Different FPER On Positive Predictive Values

		Called by assay				
		Pos.	Neg.	Total		
True	Pos.	47,500	2500	50,000	95.00%	(Sensitivity or 1-FNER)
	Neg.	47,500	902,500	950,000	*95.00%*	*(Specificity or 1-FPER)*
	Total	95,000	905,000	1,000,000		
		*50.00%*	99.72%	5.00%		
		*(PPV)*	(NPV)	(Prevalence)		

Cut point set with a 5% FPER (95% specificity). (Assuming 95% sensitivity and 5% antibody prevalence in the general population). A population size of 1 million is assumed for illustration

**Table III:** Cut Point Set With A 0.1% FPER (99.99% Specificity)

		Called by assay				
		Pos.	Neg.	Total		
True	Pos.	47,500	2500	50,000	95.00%	= (Sensitivity or 1-FNER)
	Neg.	95	949,905	950,000	*99.99%*	*= (Specificity or 1-FPER)*
	Total	47,595	952,405	1,000,000		
		*99.80%*	99.74%	5.00%		
		*(PPV)*	(NPV)	(Prevalence)		

(Assuming 95% sensitivity and 5% antibody prevalence in the general population). A population size of 1 million is assumed for illustration

One concern with targeting a very low FPER of 0.01% is that it may result in higher FNER (i.e., lower analytical sensitivity). Interestingly, in the scenario of 5% prevalence, higher FNER does not result in an appreciable decline in the negative predictive value; for example, with 20% FNER (80% sensitivity), the NPV drops only slightly to 98.96%, and PPV remains high at 99.76%, and even in the scenario of 20% antibody prevalence, the NPV drops only to 95.24%, and with 40% antibody prevalence, the NPV drops to 88.23% (Table IV). Due to the relatively greater importance of specificity over sensitivity and PPV over NPV, and due to the minor impact on NPV when targeting a very low FPER, we believe it is appropriate to set the FPER for a diagnostic cut point threshold at as low as 0.1% or even 0.01% for reliable detection of SARS-CoV-2-reactive antibodies. Given the need for setting the cut point at such a low FPER, we propose testing at least 100 presumptive antibody-negative serum samples from COVID-19-negative individuals in duplicate across six or more assay runs using a balanced block design framework during pre-study assay validation (21,24). Depending on the assay design, it would be appropriate to also evaluate potential sources of both fixed and random variation, such as analyst and instrument. The increased number of observations is needed to reliably estimate the cut point at the more extreme FPER level.

**Table IV:** Cut Point Set With A 0.1% FPER (99.99% Specificity)

		Called by assay				
		Pos.	Neg.	Total		
True	Pos.	320,000	80,000	400,000	80.00%	(Sensitivity or 1-FNER)
	Neg.	60	599,940	600,000	99.99%	(Specificity or 1-FPER)
	Total	320,060	679,940	1,000,000		
		99.98%	88.23%	40.00%		
		(PPV)	(NPV)	(Prevalence)		

A population size of 1 million is assumed for illustration

Consistent with the practice used for ADA testing, we believe that an assay’s signal responses should be normalized to accommodate analytical drift and random variation that occur naturally across plate-based assays and runs. For specific detection of anti-SARS-CoV-2 serum antibodies, our intended normalization strategy will be outlined in detail in another manuscript that is currently undergoing preparation. After the evaluation and removal of analytical and biological outliers (24), the cut point on normalized signal responses can be determined based on the calculation of mean + 3.09 × SD (standard deviation), which represents the 99.9th percentile of the population under normal distribution, and therefore corresponds to 0.1% FPER (similarly, the mean + 3.719 × SD can be used to target 0.01% FPER). To help ensure approximate data normality, this calculation can be performed after logarithmic or other suitable data transformation.

Other robust statistical alternatives to mean and SD, such as median and 1.4826 × MAD (median absolute deviation), may also be employed (24), if there is an appreciable number of borderline outliers even after eliminating more extreme outliers. Upon establishment of a screening cut point during method validation, samples from the population that have response values exceeding the screening cut point are classified as having SARS-CoV-2-reactive antibodies. Implementation of this cut point strategy will provide investigators with a statistically determined response threshold that targets a predefined FPER so the resultant cut point will be low enough to reliably include antibody-positive individuals but also sufficient to avoid misclassification of antibody-negative individuals.

In conclusion, lessons learned over the past two decades for statistical setting of screening cut points to detect reactive serum ADA for supporting clinical immunogenicity assessments of biotherapeutics will likely be beneficial for use in diagnostic assays for detection of humoral antibody responses to viruses, such as SARS-CoV-2. While the commonly used ADA-tiered testing approach is likely not practical operationally for application in a diagnostic lab setting, the strategy used for setting ADA screening cut points could be of much value in preliminary establishment of diagnostic cut points to ensure suitable assay performance for detection of SARS-CoV-2-reactive antibodies. Thus, today’s systematic data-driven approach for setting ADA cut points that includes evaluation of antibody-negative samples combined with the removal of analytical and biological outliers prior to cut point determination, like the one described herein using either a 0.1 or 0.01% FPER, is worthy of consideration for use in diagnostic assays to help control error rates prior to evaluation of clinical sensitivity and specificity and conventional analysis by a ROC plot. We believe that the application of this widely used statistical strategy for setting of ADA screening cut points could result in greater consistency among commercial diagnostic assays and LDTs for reliable detection of SARS-CoV-2 antibodies.
